# Plasma membrane transporters GAT-1 and GAT-3 contribute to heterogeneity of GABAergic synapses in neocortex

**DOI:** 10.3389/fnana.2014.00072

**Published:** 2014-07-25

**Authors:** Marcello Melone, Silvia Ciappelloni, Fiorenzo Conti

**Affiliations:** ^1^Section of Neuroscience and Cell Biology, Department of Experimental and Clinical Medicine, Università Politecnica delle MarcheAncona, Italy; ^2^Center for Neurobiology of Aging, INRCA IRCCSAncona, Italy; ^3^Foundation for Molecular Medicine, Università Politecnica delle MarcheAncona, Italy

**Keywords:** GABA, GABA transporters, GAT-1, GAT-3, symmetric synapses, heterogeneity

## Abstract

Cortical GABAergic synapses exhibit a high degree of molecular, anatomical and functional heterogeneity of their neurons of origins, presynaptic mechanisms, receptors, and scaffolding proteins. GABA transporters (GATs) have an important role in regulating GABA levels; among them, GAT-1 and GAT-3 play a prominent role in modulating tonic and phasic GABA_A_R-mediated inhibition. We asked whether GAT-1 and GAT-3 contribute to generating heterogeneity by studying their ultrastructural localization at cortical symmetric synapses using pre- and post-embedding electron microcopy. GAT-1 and GAT-3 staining at symmetric synapses showed that in some cases the transporters were localized exclusively over axon terminals; in others they were in both axon terminals and perisynaptic astrocytic processes; and in some others GAT-1 and GAT-3 were in perisynaptic astrocytic processes only. Moreover, we showed that the organizational pattern of GAT-1, but not of GAT-3, exhibits a certain degree of specificity related to the post-synaptic target of GABAergic synapses. These findings show that symmetric synapses expressing GAT-1 or GAT-3 are heterogeneous, and indicate that plasma membrane transporters can contribute to synaptic heterogeneity.

## Introduction

Heterogeneity is a hallmark of chemical synapses; this property is crucial for development of connectivity, function of neural circuits and systems, and plasticity, and has profound implications for neuropsychiatric diseases (e.g., Conti and Weinberg, 1999; Cherubini and Conti, 2001, for glutamatergic and GABAergic synapses). This view has been nicely described by O'Rourke and colleagues in the concluding paragraph of a scholarly and inspiring review: “We must recognize that uncharted synapse diversity is a scientific liability capable of severely restricting our ability to understand neural circuit function and even basic mechanisms of synapse function. Conversely, a more complete understanding of synapse diversity is certain to be a strong asset to both synapse and circuit science” (O'Rourke et al., 2012).

As far as GABAergic synapses are concerned, heterogeneity has been demonstrated at all levels so far studied: from morphology and chemical phenotype of their neurons of origin to presynaptic mechanisms, from ionotropic and metabotropic pre- or post-synaptic receptors to anchoring proteins, and from post-synaptic responses to plasticity phenomena (e.g., Aradi et al., 2002; Soltesz, 2005; Maffei, 2011; Méndez and Bacci, 2011; Sassoè-Pognetto et al., 2011; Fritschy et al., 2012; O'Rourke et al., 2012; Benarroch, 2013; Bragina et al., 2013; DeFelipe et al., 2013).

Since Iversen and colleagues demonstrated the existence of a high-affinity uptake of exogenous GABA by a subpopulation of cortical axon terminals (Iversen and Neal, 1968; Bloom and Iversen, 1971), much has been learnt on the nature, distribution, mechanisms, and functional role of the proteins mediating GABA uptake in neocortex (GABA transporters, GATs) (Borden, 1996; Conti et al., 2004; Richerson and Wu, 2004; Héja et al., 2006; Kanner, 2006; Kristensen et al., 2011; Pramod et al., 2013). Yet, the possible contribution of GATs to GABAergic synapses heterogeneity has never been subjected to experimental scrutiny. Here, we address this issue and suggest that GATs add to the long list of proteins generating heterogeneity at GABAergic synapses.

## GABA transporters in cerebral cortex

Four GATs have been identified to date: GAT-1 (slc6a1), GAT-2 (slc6a13), GAT-3 (slc6a11), and BGT-1 (slc6a12) (Borden, 1996; Conti et al., 2004). GATs share a high degree of nucleotide and amino acid sequence homology; they transport GABA in a high affinity, Na^+^ and Cl^−^ dependent manner, but they differ in their tissue distribution and pharmacological properties (Madsen et al., 2007).

GAT-1 is localized to axon terminals (AT) forming symmetric synapses and to astrocytic processes (AP) (Radian et al., 1990; Minelli et al., 1995; Conti et al., 1998); a recent analysis showed that in parietal cortex ~55% of GAT-1 is in neuronal elements, and ~40% is in AP; and that ~60% of all GAT-1 is in profiles contributing to synapses (Melone et al., 2013). Accordingly, GAT-1 has a prominent role in both tonic and phasic GABA_A_R-mediated inhibition, particularly during sustained neuronal activity (Bragina et al., 2008); GAT-1 also contributes to presynaptic homeostasis at GABAergic terminals (Conti et al., 2011). GAT-1 is strongly inhibited by *cis*-3-aminocyclohexane carboxylic acid (ACHC) and, to a lesser extent, by 2, 4 diaminobutyric acid, but not by β-alanine (Madsen et al., 2007). GAT-1 developmental expression is coordinated with that of other GABAergic presynaptic proteins, i.e., the synthesizing enzyme GAD and the vesicular transporter VGAT, and parallels that of the GABA_A_ receptor α1 subunit, which participates in mature GABAergic transmission (Minelli et al., 2003a; Conti et al., 2004 for data and references). GAT-2 is mainly expressed in leptomeninges and in ependymal and choroid plexus cells (Conti et al., 1999); its function is still elusive. GAT-3 is localized to distal AP (~70%) and to some AT (~25%); about half of all GAT-3 is localized in profiles contributing to synapses (Minelli et al., 1996; Melone et al., 2013). The functional role of GAT-3 has not been definitely clarified, although it is believed to modulate the amount of GABA diffusing into extracellular space (Conti et al., 2004; Kersanté et al., 2013; Melone et al., 2013). GABA uptake by GAT-2 and GAT-3 is inhibited by β-alanine, but not by ACHC (Madsen et al., 2007). In neonatal cortex, only GAT-3 is abundantly expressed and GABA uptake is potently inhibited by β-alanine, suggesting that extracellular GABA levels at birth are modulated mainly by GAT-3 (Minelli et al., 2003b). Interestingly, phylogenetic studies show that GAT-1 precedes GAT-3 during evolution (Kinjo et al., 2013). As far as BGT-1 is concerned, it is unclear whether this transporter functions as a GAT in CNS (Lehre et al., 2011).

## Localization of GAT-1 and GAT-3 at cortical synapses is heterogeneous

With this background, we verified the possibility that symmetric synapses have different expression patterns of GATs. We focused on GAT-1 and GAT-3, which are expressed at synapses and affect synaptic transmission (Section GABA Transporters in Cerebral Cortex). All observations were from layers II/III of the first somatic sensory cortex of the parietal lobe.

**Figure 1:**
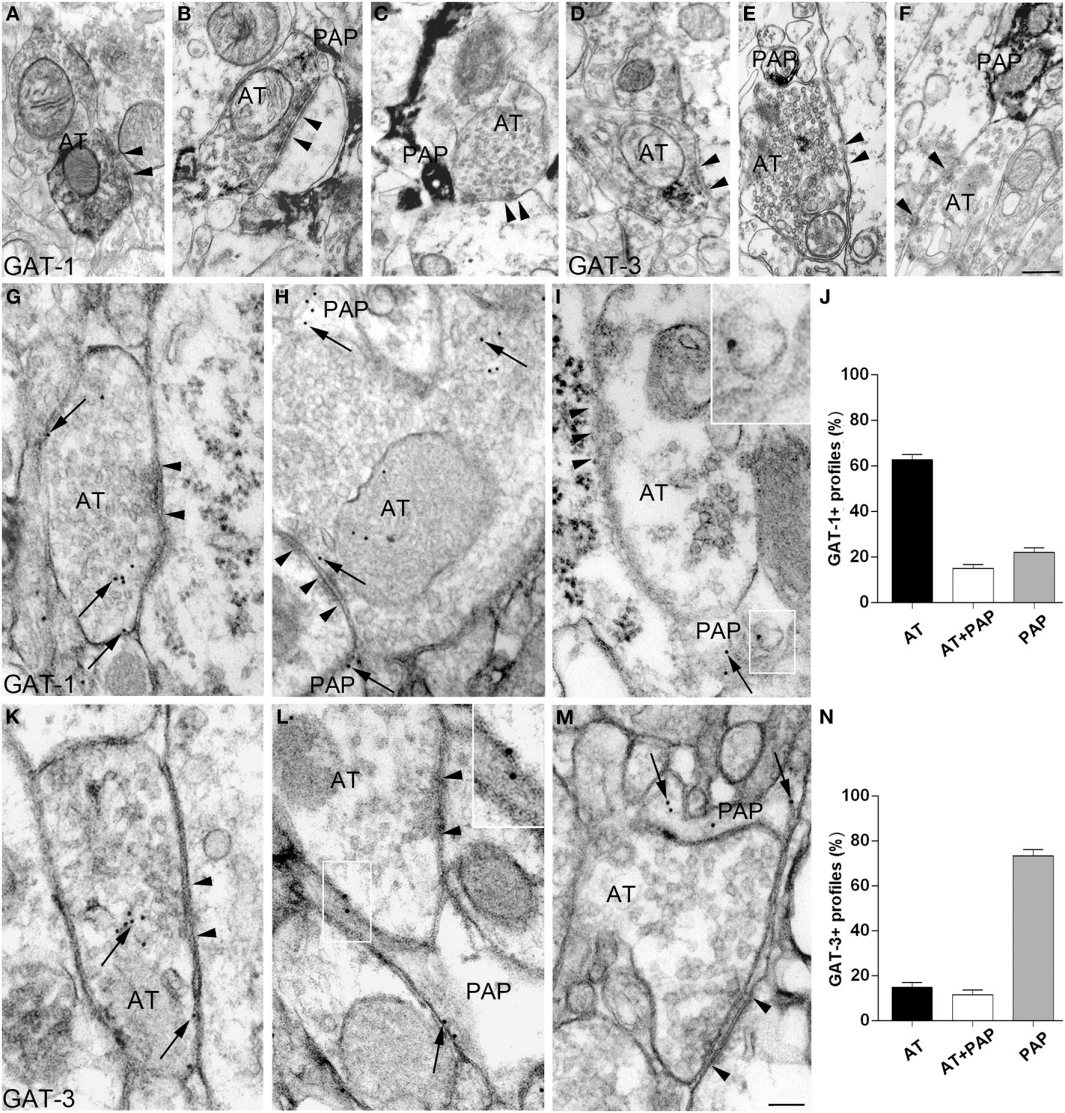
**Heterogeneous distribution of GATs at cortical symmetric synapses (layer II/III of the first somatic sensory cortex)**. **(A–F)** GAT-1 and GAT-3 immunoreactivities at symmetric synapses in pre-embedded material. Electron microscopic inspection revealed that GAT-1 **(A–C)** and GAT-3 **(D–F)** immunoreactivities are localized either to axon terminals (AT) only [**(A)** for GAT-1; **(D)** for GAT-3], or to both AT and perisynaptic astrocytic processes (PAP) [**(B)** for GAT-1; **(E)** for GAT-3], or exclusively to PAP surrounding synapses [**(C)** for GAT-1; **(F)** for GAT-3]. Arrowheads point to symmetric synaptic contacts. Pre-embedding was performed as described (Melone et al., 2013) in sections from 3 animals/antigen. **(G–N)** GAT-1 **(G–I)** and GAT-3 **(K–M)** staining in AT making symmetric synaptic contacts and in PAP. Framed regions in **(I,L)** are reproduced, enlarged, in the upper right corner. Arrows indicate both cytoplasmic and membrane-associated GAT-1 or GAT-3 staining and arrowheads point to symmetric synaptic contacts. Post-embedding was performed by osmium-free embedding method (Phend et al., 1995) as described (Melone et al., 2013) on sections from four rat brains. Profiles were considered immunopositive for GAT-1 or GAT-3 when gold particle density was significantly higher than background, estimated by calculating gold particle density over pyramidal cell nuclei (Supplementary Table 1). Scale bars: 100 nm.

We first analyzed qualitatively the organization of synapses expressing GAT-1 and GAT-3 using a pre-embedding method. This analysis showed that, at symmetric synapses, GAT-1 (Figures 1A–C) and GAT-3 (Figures 1D–F) were localized either in AT or in both perisynaptic astrocytic processes (PAP) and AT, or in PAP. Next, we used an immunogold post-embedding method to visualize GATs molecules inserted in membranes (and therefore conceivably functional). Densities of GAT-1- and GAT-3 in background, AT, and PAP are given in the Supplementary Table 1. Analysis of the distribution of GAT-1 staining at symmetric synapses (*n* = 462) showed that 62.7 ± 2.2% of GAT-1+ profiles were AT; 15.1 ± 1.6% both AT and PAP; and 22 ± 1.9% PAP (Figures 1G–J). Analysis of GAT-3 staining at symmetric synapses (*n* = 249) revealed that 73.4 ± 2.8% of positive profiles were PAP; 14.8 ± 2.2% AT; and 11.6 ± 2% both PAP and AT (Figures 1K–N). Thus, symmetric synapses expressing GAT-1 or GAT-3 are indeed heterogeneous, as some of them express a GAT only in AT, others only in PAP, and some others in the two synaptic elements.

**Figure 2:**
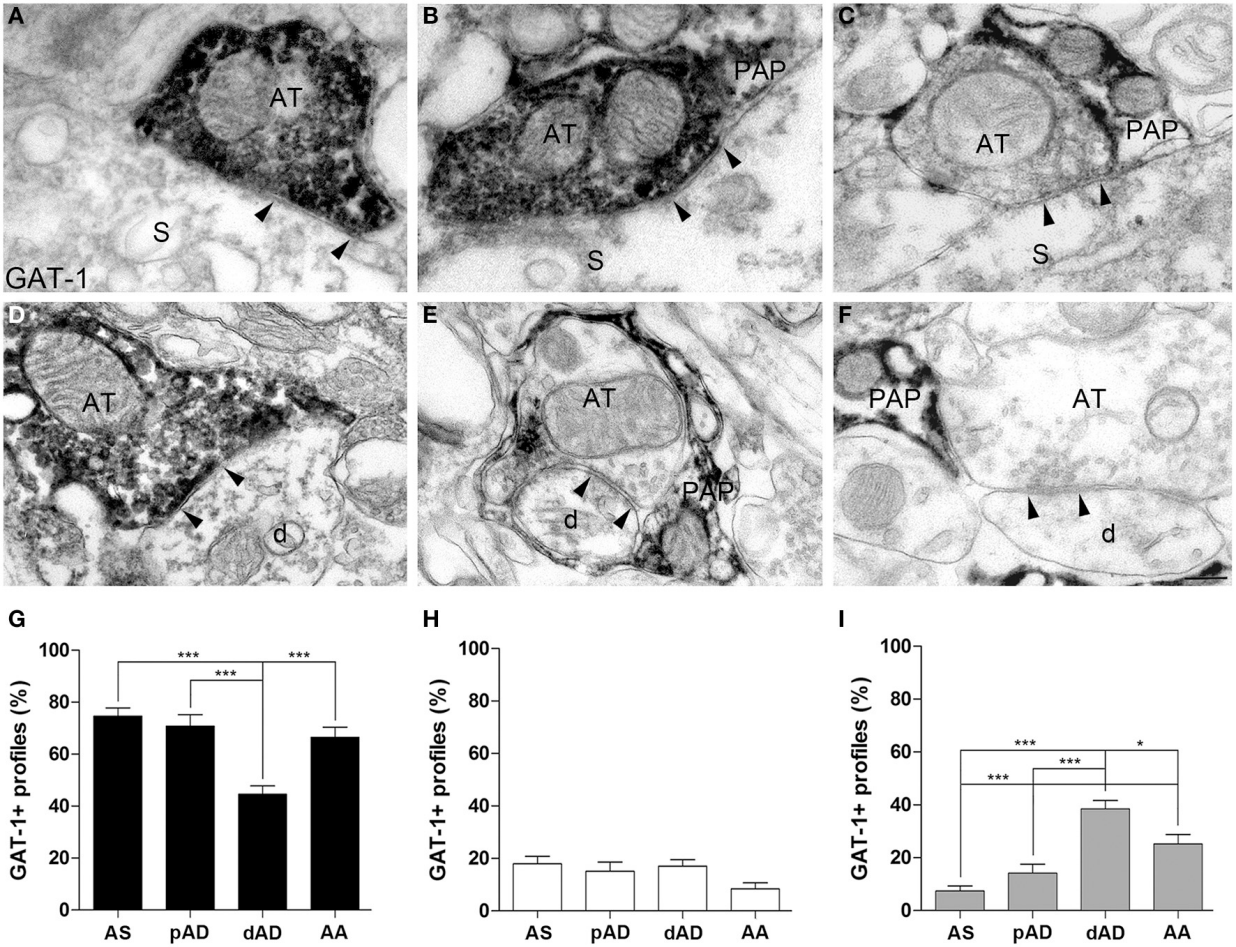
**Distribution of GAT-1 at axo-somatic (AS), proximal axo-dendritic (pAD), distal axo-dendritic (dAD) and axo-axonic (AA) synapses. (A–F)** Examples of GAT-1 immunoreactivity at AS **(A–C)** and dAD **(D–F)** symmetric synapses in which GAT-1 was localized at AT only **(A,D**), at both AT and PAP **(B,E)**, or at PAP only **(C,F)**. Arrowheads point to symmetric contacts. AT, axon terminal, PAP, perisynaptic astrocytic processes; S, soma; d, distal dendrite. **(G–I)** Quantification of GAT-1+ profiles at AS, pAD, dAD, and AA synapses. Black columns refer to synapses in which GAT-1 was only in AT, white columns to synapses in which it was in both AT and PAP, and gray columns to synapses where GAT-1 was only in PAP. ^*^P < 0.05; ^***^P < 0.001. Scale bar: 100 nm.

GABAergic synapses can be differentiated on the basis of post-synaptic targets (e.g., Somogyi et al., 1998; DeFelipe et al., 2002; Ascoli et al., 2008). We therefore investigated whether the three organizational models (GAT in AT only, in PAP only, and in both) showed differences between axo-somatic, axo-dendritic, and axo-axonic GABAergic synapses onto pyramidal neurons using pre-embedding electron microscopy. We studied 189 axo-somatic (AS), 146 proximal axo-dendritic (pAD), 229 distal axo-dendritic (dAD) (dendrites were considered distal if their diameter was < or = 1 μm, proximal if it was > 1 μm), and 173 axo-axonic (AA) GAT-1+ synapses. ANOVA analysis showed that: (1) synapses in which GAT-1 was only in AT differed between AA, pAD, AA (74.6 ± 3.1, 70.7 ± 4.4, 66.4 ± 3.9%, in the order), and dAD (44.5 ± 3.2%) synapses (Figures 2A,D,G); (2) synapses in which GAT-1 was both in AT and in PAP did not differ between groups (17.9 ± 2.8, 15 ± 3.4, 17 ± 2.4, 8.3 ± 2.3% for AS, pAD, dAD, and AA synapses; Figures 2B,E,H); and (3) synapses in which GAT-1 was only in PAP differed between AS, pAD, AA (7.4 ± 1.9, 14.1 ± 3.4, 25.1 ± 3.6%, in the order) and dAD (38.4 ± 3.2%) synapses (Figures 2C,F,I), as well as between AS and AA synapses (Figure 2I). ANOVA analysis of AS (*n* = 163), pAD (*n* = 134), dAD (*n* = 150), and AA (*n* = 147) GAT-3+ synapses did not reveal any difference between groups of synapses (Supplemental Figure 1).

## Conclusion(s)

In adult cortical GABAergic synapses GAT-1 and GAT-3 are in both neuronal and astrocytic processes: GAT-1 is prevalently segregated in neuronal elements and in profiles contributing to synapses, whereas GAT-3 is mostly expressed in astrocytes and does not exhibit a preferential distribution in elements contributing to synapses (Minelli et al., 1995, 1996; Melone et al., 2013). This study showed that: (1) regardless of the important differences summarized above, GAT-1 and GAT-3 exhibit the same organizational pattern at cortical GABAergic synapses. Interestingly, the same pattern has been described for GLT-1, the major glutamate transporter, in neocortex (Melone et al., 2009); (2) from the transporter perspective, symmetric synapses can be subdivided in those expressing GAT-1 or GAT-3 in AT only, in PAP only, and in both synaptic elements; and (3) GAT-1 (but not GAT-3) organization pattern exhibit a further level of heterogeneity, related to the post-synaptic target. This indicates that GAT-1 and GAT-3 can generate a considerable degree of heterogeneity at cortical GABAergic synapses.

Although, the degree of post-synaptic specificity of GABAergic interneurons on pyramidal neurons is not absolute, several generalization can be made: AS synapses are prevalently formed by small basket cells, pAD synapses from large basket cells, dAD synapses from double-bouquet cells, and AA synapses from chandelier cells (Somogyi et al., 1998; DeFelipe et al., 2002; Ascoli et al., 2008). This would indicate that at synapses formed by double-bouquet cells GAT-1 mediated GABA uptake is more dependent on astrocytes than at synapses formed by basket and chandelier cells. It is worth noting that double bouquet cell-evoked IPSPs, recorded in pyramidal cell somata, have a smaller amplitude than those evoked by both small and large basket cells (Tamás et al., 1997). Whether this physiological features is related to the peculiar organization of the GAT-1 mediated GABA uptake system is a stimulating challenge for future studies. Furthermore, present results indicate also that at AA synapses formed by chandelier cell axons the role of astrocytic GAT-1 mediated GABA uptake may be more important than at AS synapses formed by small basket cell axons.

Overall, data reported highlight a novel aspect of GAT-1 and GAT-3 localization at cortical GABAergic synapses, and suggest that this may be a fertile field for increasing our understanding of GABAergic synapses heterogeneity.

### Conflict of interest statement

The authors declare that the research was conducted in the absence of any commercial or financial relationships that could be construed as a potential conflict of interest.

## Supplementary material

Supplemental Figure 1 **Distribution of GAT-3 at axo-somatic (AS), proximal axo-dendritic (pAD), distal axo-dendritic (dAD), and axo-axonic (AA) synapses**. In **(A**) staining is in AT only, in **(B)** at both AT and PAP, and in **(C)** in PAP only. AT, axon terminal; PAP, perisynaptic astrocytic processes; S, soma of pyramidal neuron; d, distal dendrite. **(D–E)** Quantification of GAT-3+ profiles at different synapses. Black columns refer to synapses in which GAT-3 was only in AT, white columns to synapses in which GAT-3 was in both AT and PAP, and gray columns to synapses where GAT-3 was only in PAP. Scale bar: 100 nm.
