# Supplementary figures and images for: Plasma membrane transporters GAT-1 and GAT-3 contribute to heterogeneity of GABAergic synapses in neocortex
Source: Front Neuroanat. 2014 Jul 25;8:72. doi: 10.3389/fnana.2014.00072 (PMC4110517; doi:10.3389/fnana.2014.00072)

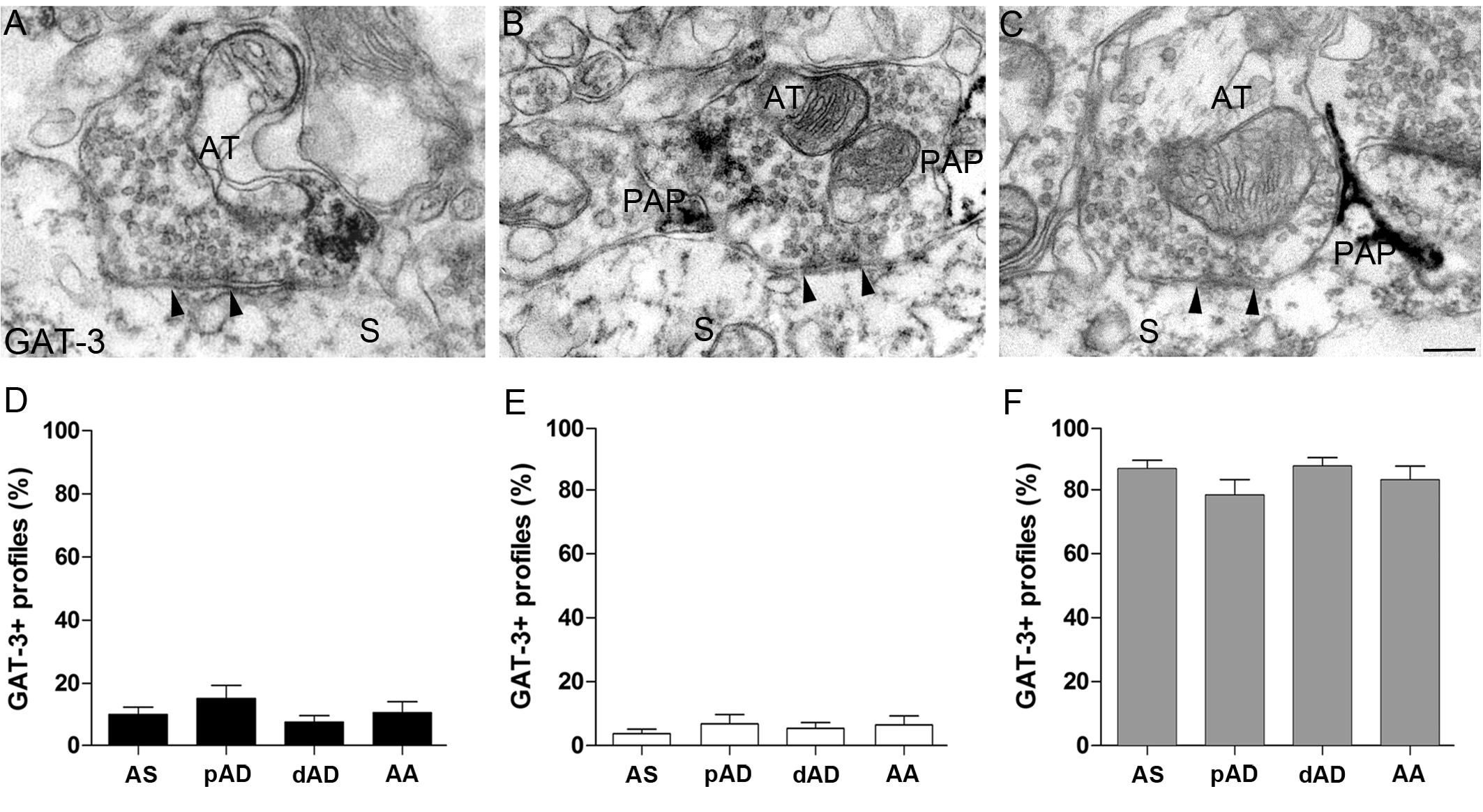

Supplement: Supplemental Figure 1 — Distribution of GAT-3 at axo-somatic (AS), proximal axo-dendritic (pAD), distal axo-dendritic (dAD), and axo-axonic (AA) synapses. In (A) staining is in AT only, in (B) at both AT and PAP, and in (C) in PAP only. AT, axon terminal; PAP, perisynaptic astrocytic processes; S, soma of pyramidal neuron; d, distal dendrite. (D–E) Quantification of GAT-3+ profiles at different synapses. Black columns refer to synapses in which GAT-3 was only in AT, white columns to synapses in which GAT-3 was in both AT and PAP, and gray columns to synapses where GAT-3 was only in PAP. Scale bar: 100 nm. [file SupplementaryFigure1.TIF]
