# Supplementary material for: Plasma membrane transporters GAT-1 and GAT-3 contribute to heterogeneity of GABAergic synapses in neocortex
Source: Front Neuroanat. 2014 Jul 25;8:72. doi: 10.3389/fnana.2014.00072 (PMC4110517; doi:10.3389/fnana.2014.00072)
Supplement: Supplementary file 2 [file Table1.DOCX]

| **Localization** | **GAT-1**  (particles/µm^2^) | **GAT-3**  (particles/µm^2^) |
| --- | --- | --- |
| Nucleus (background) | 0,61 ± 0,04 (n=36) | 0,54 ± 0,05 (n=36) |
| Axons terminalº | 6,44 ± 0,39 (n=332) | 3,67 ± 0,52 (n=57) |
| Plasma membrane§ | 20,93 ± 1,22 | 14,89 ± 1,83 |
| Cytoplasmºº | 3,88 ± 0,34 | 2,18 ± 0,42 |
| Perisynaptic astrocytic processesº | 50,36 ± 5,69 (n=243) | 29,93 ± 3,43 (n=173) |
| Plasma membrane§ | 68,92 ± 6,24 | 48,75 ± 4,23 |
| Cytoplasmºº | 17,17 ± 2,23 | 15,53 ± 3,92 |

**Table 1.** Density of GAT-1 and GAT-3 staining in axon terminals and perisynaptic astrocytic processes of symmetric synapses

Density values are mean ± SEM; n=number of profiles; two sided t-test was used for statistical analysis. GAT-1: º density of gold particles was significantly higher than background in axon terminals forming symmetric synapses and in astrocytic processes surrounding symmetric synapses (*P*<0.001 and *P*<0.01 respectively). § Density of membrane-associated particles was significantly higher than background in axon terminals and astrocytic processes (*P*<0.001). ºº Density of cytoplasmic particles was significantly higher than background in axon terminals and in astrocytic processes (*P* <0.01). GAT-3: º density of gold particles was significantly higher than background in both axons terminals and astrocytic processes (*P*<0.01). § Density of membrane-associated particles was significantly higher than background in axon terminals and astrocytic processes (*P*< 0.001). ºº Density of cytoplasmic particles was significantly higher than background in axon terminals and in distal astrocytic processes (*P*< 0.01).
